# Neurocognition and functioning in adolescents at clinical high risk for psychosis

**DOI:** 10.1186/s13034-023-00567-1

**Published:** 2023-02-08

**Authors:** Martina Maria Mensi, Marika Orlandi, Erica Casini, Ana Catalan, Gonzalo Salazar de Pablo, Paolo Fusar-Poli, Renato Borgatti

**Affiliations:** 1 Child Neurology and Psychiatry Unit, IRCCS Mondino Foundation, Via Mondino 2, 27100 Pavia, Italy; 2 Psychiatry Department, Facultad de Medicina y Odontología, Centro de Investigación en Red de Salud Menta (CIBERSAM), Biocruces Bizkaia Health Research Institute, OSI Bilbao-Basurto, University of the Basque Country UPV/EHU, Instituto de Salud Carlos III, Barakaldo, Bizkaia Spain; 3 Early Psychosis: Interventions and Clinical-Detection (EPIC) Lab, Department of Psychosis Studies, Institute of Psychiatry, Psychology & Neuroscience, King’s College London, London, UK; 4 Department of Child and Adolescent Psychiatry, Institute of Psychiatry, King’s College London, Psychology & Neuroscience, London, UK; 5 Child and Adolescent Mental Health Services, South London and Maudsley NHS Foundation Trust, London, UK; 6 Department of Child and Adolescent Psychiatry, Institute of Psychiatry and Mental Health, Hospital General Universitario Gregorio Marañón School of Medicine, Universidad Complutense, Instituto de Investigación Sanitaria Gregorio Marañón (IiSGM), CIBERSAM, Madrid, Spain; 7 Maudsley Biomedical Research Centre, National Institute for Health Research, South London and Maudsley NHS Foundation Trust, London, UK; 8 OASIS Service, South London and Maudsley NHS Foundation Trust, London, UK; 9 Department of Brain and Behavioural Sciences, University of Pavia, Pavia, Italy

**Keywords:** Adolescence, Clinical high risk for psychosis, Functioning, Neurocognition, Psychosis

## Abstract

**Background:**

Once psychosis has set in, it is difficult for patients to achieve full recovery. Prevention of psychosis and early intervention are promising for improving the outcomes of this disorder. In the last two decades, neurocognition has been studied as a biomarker for clinical-high risk for psychosis (CHR-P). However, neurocognitive functioning has been under-investigated in adolescents.

**Methods:**

We enrolled 116 adolescents from 12 to 17 years old (*mean* = 15.27, *SD* = 1.56; 76 females). This 3-year cohort study aimed to identify differences in neurocognitive and overall functioning in three groups of adolescent patients divided according to the semi-structured interview Comprehensive Assessment of At-Risk Mental States (CAARMS): adolescents with established psychosis, adolescents with CHR-P, and adolescents not meeting either criteria (non-CHR-P). To differentiate the profiles, clinicians administered cognitive evaluation and neuropsychological tasks. Moreover, they filled in scales to assess their global, social, and role functioning and a questionnaire to assess the severity of the disease.

**Results:**

We made a between-group comparison on neurocognitive measures and found that the CHR-P and the psychosis groups differed in processing speed (TMT-A;* p* = .002 in BVN categorial fluency (*p* = .018), and Rey–Osterrieth complex figure drawing from memory task (*p* = .014), with psychosis group showing worse performance. No differences emerged between non-CHR-P and CHR-P (*p* = .014) individuals. CHR-P had better functioning than the psychosis group but worse than the non-CHR-P one.

**Conclusions:**

These results confirm that neurocognition can be a helpful biomarker in identifying specific subgroups of adolescents with emerging psychopathology and help clinicians develop stratified preventive approaches.

**Supplementary Information:**

The online version contains supplementary material available at 10.1186/s13034-023-00567-1.

## Introduction

Psychotic disorders typically have their onset in adolescence and early adulthood, with the peak of the risk occurring between the ages of 12 and 25 years [1]. After the onset of the disorder, it is challenging to improve its course and lead the patient to complete recovery [2, 3]. Therefore, prevention of psychosis and early intervention are promising paths for improving outcomes [4]. In light of the above, in the last twenty years, attention to prevention has focused on the clinical-high risk for psychosis (CHR-P) population. CHR-P population includes three subgroups: Attenuated psychotic Syndrome (APS), Brief intermittent psychotic symptoms (BLIPS), and Genetic risk and Deterioration Syndrome (GRD) [5]. Several studies have highlighted the importance of detection, prognosis, and interventions for CHR-P individuals and the formulation of updated recommendations, mainly because detection of CHR-P individuals is based on patients’ referral, and symptoms may remain undetected for a long time [6]. So, childhood and adolescence represent a critical developmental window where opportunities to gain social and adaptive abilities depend on the individuals' neurocognitive performance [1]. Therefore, early intervention and particularly preventive approaches in young people with subtle signs and symptoms of the psychotic disorder (termed ‘primary indicated prevention’ [4, 7]) have the potential to benefit the lives of many young people.

Although the CHR-P prevention paradigm is particularly promising, especially in young people, empirical challenges arise [8]. Researchers stated that neurocognition could be a biomarker that may help professionals distinguish CHR-P from health controls (HC) and could help determine the risk of transition to psychosis. In this connection, a recent meta-analysis [9] comparing a total of 78 independent studies with 5162 CHR-P individuals and 2865 HC described that the first group showed medium to large deficits in the studied neurocognitive domains. Moreover, CHR-P people were less impaired than individuals with a first episode of psychosis. Knowing the global functioning and performance trends of CHR-P patients on neuropsychological tests can also help clinicians intervene early to reduce the risk of transition to psychosis, which is currently relevant in the adolescent population [10, 11].

Despite this recent work, there is not much evidence that synthesizes current knowledge about neurocognitive functioning in adolescent individuals [12–17], specifically about longitudinal changes across time in this population [13, 17]. Moreover, as shown in the metanalysis [9], studies in adolescence show different results because of different tasks used, non-homogeneous samples, or severe comorbid disorders [8, 17]. Indeed, it is crucial to find biological and psychological markers of transition to psychosis to help clinicians detect psychotic symptoms, prevent psychotic disorders, and formulate a prognosis to offer the most appropriate interventions. Overall, the empirical literature on the neurocognitive performance of children and adolescents is poorer in comparison with the one on young adults, so there is a gap in the literature.

In light of that, this study aimed to identify differences in neurocognitive functioning and overall functioning in three groups of adolescent patients divided according to their emerging psychopathology ascertained through the semi-structured interview Comprehensive Assessment of At-Risk Mental States (CAARMS) criteria [18]: i) Psychosis, ii) CHR-P, and iii) non-CHR-P.

We expected to find worse performance in neurocognitive tasks and lower functioning in the psychosis group, moderate deficits in the CHR-P group, and average performances and adequate global functioning in the non-CHR-P group.

## Methods

### Study design

We planned a 3-year cohort study, previously described in the literature [19], conducted according to the Reporting of studies Conducted using the Observational Routinely collected health Data (RECORD) statement (see Additional file 1). The study received the approval of the Ethics Committee of Policlinico San Matteo in Pavia, Italy (P-20170028892). The authors assert that all procedures contributing to this work comply with the Helsinki Declaration of 1964 and its later amendments and with the ethical standards of the relevant national and institutional committees on human experimentation. The dataset is available upon request in Zenodo [20].

### Sample

We enrolled 116 participants who have been referred to the Child Neurology and Psychiatry Unit of the third-level Scientific Hospitalization and Treatment Institution (IRCCS) Mondino Foundation in Pavia from 2017 to 2020. Mondino Foundation is a clinical and research institute; where many workers also have a role within the University. Cinical practice is almost always carried out together with research, so the opportunity to participate in research protocols is well received by patients and families. Specifically, the Child Neurology and Psychiatry Unit is a department with several teams of physicians and psychologists. Our group focuses on the psychiatry branch and is specialized in the diagnosis and care of patients with serious psychopathological diseases.

As stated in the original protocol [19], we included in the study help-seeking male and female inpatients adolescents between 12 and 17 years of age from all over Italy and taken care of for psychiatric disorders at Child Neurology and Psychiatry Unit and who had provided, together with their parents or guardians, their written informed consent.

We excluded participants who had a history of psychosis according to DSM-5 criteria before assessment, who had head injuries or any other underlying medical/neurological condition that could explain psychiatric symptoms, who had a current DSM-5 illicit substance addiction or induced mental disorders, who presented intellectual disability (IQ ≤ 70) assessed through WISC-IV [21] or WAIS-IV [22], or whose parents declined participation or did not provide written informed consent.

To homogenize the CHR-P group, we excluded adolescents who met the CAARMS [18] criteria for the vulnerability group, i.e., with a combination of a trait risk factor and significant functioning impairment, and those who met Brief Limited Intermittent Psychotic Symptoms (BLIPS) group criteria given the phenotypic overlap of this subgroups with the psychosis one.

**Fig. 1:**
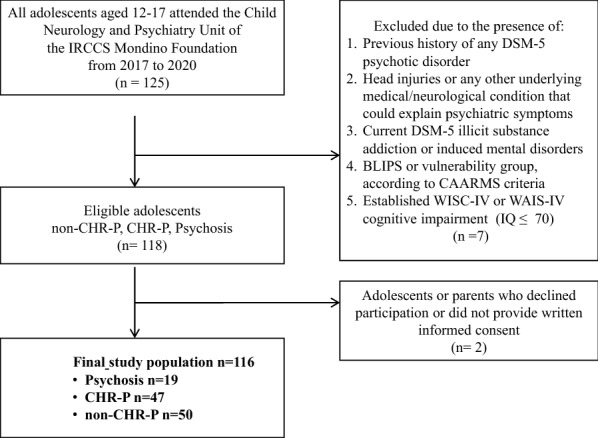
Flowchart of the study sample

We divided eligible patients into three groups according to the semi-structured interview CAARMS, a valuable tool to be integrated into diagnostic assessment in Child Neuropsychiatry services [23–26]. The three groups were (i) Psychosis, including adolescents who received over-threshold scores for the CAARMS psychosis group; (ii) CHR-P, including adolescents who both met the criteria for Attenuated psychotic Syndrome (APS) according to the DSM-5 [27] and received suprathreshold scores for the CAARMS CHR-P groups (i.e., intensity or frequency) (in our sample the CHR-P group overlapped the definition of APS); (iii) non-CHR-P, including patients who did not meet the CAARMS criteria for psychosis group nor CHR-P groups*. Although* the third group not including healthy controls, we referred to previous studies that used a sample of subjects with different diseases of milder severity than the patient group (e.g., headache, learning disabilities, internalizing problems) [28, 29]. An appropriately trained psychologist or neuropsychiatrist on the CAARMS administered the interview. For cases in which there were doubts, the assessor compared with an expert colleague and the score was given following their discussion. Figure 1 shows the study population flowchart, and Table 2 shows the patients' diagnoses for each group in detail.

### Instruments

A trained psychologist thoroughly explained the study to families. A clinician collected sociodemographic information, previous medical and psychiatric history, socio-economic status (SES) [30], and family history of any DSM-5 psychiatric disorders. A child neuropsychiatrist or a psychologist administered the Wechsler scale to exclude intellectual disability and then conducted the standardized clinical interview Kiddie Schedule for Affective Disorders and Schizophrenia—Present and Lifetime Version (K-SADS-PL) for DSM-5 [31, 32] with the participants and their parents or guardians separately, to confirm the diagnosis. All diagnoses were made according to DSM-5 criteria [27] and confirmed using K-SADS-PL. To assess symptoms attributable to personality disorders and structuring personality disorders, we administered the Structured Clinical Interview for DSM-5 Personality Disorders (SCID-5 PD) [33] to patients aged 14 and over.

A trained psychologist administered an in-depth neuropsychological assessment focusing on several neurocognitive domains to assess the neurocognitive profile. To assess the intelligence quotient (IQ), we administered the Wechsler intelligence scale (WISC-IV or WAISIV) [21, 22, 34]. To assess visuospatial planning and attention, we administered Rey–Osterrieth complex figure test (ROCF) [35–37], and to evaluate processing speed and executive functioning, we administered the Trail Making Test Part A (TMT-A) and B (TMT-B), both measuring processing speed [38, 39] and TMT-B executive functioning. Moreover, we used many of the subtests contained in the BVN 12–18 (Batteria per la Valutazione Neuropsicologica dell’Adolescenza—Adolescent Neuropsychological Assessment Battery) [40] to assess lexical denomination, verbal working memory (forward and backward digit span), nonverbal working memory (Corsi Block-tapping test), selective auditory and visual attention, phonemic and categorial fluency, and reasoning and problem-solving (Elithorn Perceptual Maze).

To evaluate the level of functioning, clinicians compiled the Children’s Global Assessment Scale (CGAS) [41] and the Social and Occupational Functioning Assessment Scale (SOFAS) [42]. We also compiled the Global Functioning: Role scale (GF:R) (Niendam et al. [43]) and Global Functioning: Social scale (GF:S) [44]. Clinicians assessed the overall severity of illness using the Clinical Global Impression-Severity (CGI-S) scale [45, 46].

### Statistical methods

Descriptive analyses were performed for demographic and clinical characteristics, for the total sample, and separately for each of the three groups (Psychosis vs. non-CHR-P vs. CHR-P). These analyses included mean value and standard deviation (SD), as appropriate for continuous variables, and absolute and relative frequencies for categorical variables. Statistical comparisons between the three groups completed descriptive analyses. Given the small sample size, Kruskal–Wallis was used for numerical variables (i.e., age), complemented by post hoc analyses (Dunn test), and the Fisher’s exact test for categorical variables (i.e., gender, ethnicity, and SES). To reduce the chance of type I error due to multiple testing, Bonferroni correction was applied to all post hoc analyses. We then performed a between-group comparison on neurocognitive measures (i.e., WISC-IV/WAIS-IV, TMT-A, TMT-B, BVN subtests, and ROCF). Since our groups were non-equal in size, we used the Kruskal–Wallis test, complemented by post-hoc Dunn's test with a Bonferroni correction. Statistical analyses were performed with IBM SPSS version 27.0 [47].

## Results

### Participants

The sample comprised 116 adolescents aged between 12 and 17 years old. Figure 1 shows the study population flowchart.

Considering the whole sample, 26 adolescents (22.4%) came from low socio-economic status (SES) families, 26 (22.4%) came from low-to-medium–low SES families, 36 adolescents (31.0%) from medium SES families, 18 (15.5%) from medium-to-high SES families, and 6 (5.2%) from high SES families.

At baseline, 19 out of 116 (16.4%) met the CAARMS criteria for psychosis, 47 (40.5%) met the criteria for CHR-P, and 50 (43.1%) met neither criterion. Table 1 shows sociodemographic information and family history of psychiatric disorders in the total sample and the three subgroups.

**Table 1:** Sociodemographic data and family history of psychiatric disorders in the total sample and the three subgroups

Characteristic	Total (N = 116)	Non-CHR-P (N = 50)	CHR-P (N = 47)	Psychosis (N = 19)	p*
Sociodemographic					
Age, mean (SD), y	15.27 (1.56)	15.4 (1.60)	15.3 (1.46)	14.85 (1.72)	0.497
Sex, female, n (%)	76 (65.0)	34 (68)	32 (68.1)	10 (52.6)	0.492
Ethnicity, n (%)					0.292
Italian	93 (80.2)	38 (76)	37 (78.7)	18 (94.7)	
Hispanic	1 (0.9)	1 (2.0)	0 (0)	0 (0.0)	
Eastern European	7 (6.0)	4 (80)	3 (6.4)	1(5.3)	
African	3(2.6)	0 (0.0)	2 (4.3)	1 (5.3)	
Other	9 (7.8)	6 (12)	3 (6.4)	0 (0.0)	
Socio Economic Status, median (IQR25, 75)	30.75 (19.6, 38.9)	30.25 (20.2, 39.0)	30.0 (17.5, 38.7)	32 (22.0, 37.0)	0.789
Adopted, n (%)	5 (4.3)	3 (6.0)	2 (4.3)	0 (0.0)	0.842
Separated-divorced parents, n (%)	41 (35.3)	21 (42.0)	16 (34.0)	4 (21.1)	0.242
Family history of any DSM-5 psychiatric disorders, n (%)					
None	39 (33.6)	15 (30.0)	19 (40.4)	5 (26.3)	0.394
Psychosis	11 (9.5)	3 (6)	3 (8.5)	4 (21.1)	0.248
first degree	2 (1.7)	0 (0)	1 (2.1)	1 (5.3)	
second degree	9 (7.8)	3 (6)	3 (6.4)	3 (15.8)	
Depression	39 (23.6)	16 (32.0)	14 (29.8)	9 (47.4)	0.747
first degree	21 (18.1)	9 (18.0)	7 (14.9)	5 (26.3)	
second degree	18 (15.5)	7 (14.0)	7 (14.9)	4 (21.1)	
Anxiety	24 (20.7)	11 (22.0)	10 (21.2)	3 (15.8)	0.364
first degree	17 (14.7)	7 (14.0)	9 (19.1)	1 (5.3)	
second degree	7 (6.0)	4 (8.0)	1 (2.1)	2 (10.5)	
Substance abuse	10 (9.0)	3 (6.0)	5 (10.6)	2 (10.6)	0.619
first degree	19 (8.0)	3 (6.0)	4 (8.5)	1 (5.3)	
second degree	8 (6.9)	0 (0.0)	1 (2.1)	1 (5.3)	
Disruptive disorder	3 (2.6)	1 (2.0)	1 (2.1)	1 (5.3)	0.368
first degree	1 (0.9)	0 (0.0)	1 (2.1)	0 (0.0)	
second degree	2 (1.7)	1 (2.0)	0 (0.0)	1 (5.3)	
Eating disorder	3 (2.6)	2 (4.0)	1 (2.1)	0 (0.0)	1.00
first degree	2 (1.7)	1 (2.0)	1 (2.1)	0 (0.0)	
second degree	1 (0.9)	1 (2.0)	0 (0.0)	0 (0.0)	
Other	20 (25.9)	12 (24.0)	12 (25.5)	6 (29.6)	0.617
first degree	8 (6.9)	3 (6.0)	4 (8.5)	1 (5.3)	
second degree	22 (19.0)	9 (18.0)	8 (17.0)	5 (26.3)	

Table 2 shows patients’ history of psychiatric disorders, psychopathology, global functioning, and baseline exposure to psychiatric treatments in the sample and the subgroups. Additional file 2: Table S1 shows post-hoc analyses.

**Table 2:** Personal history of psychiatric disorders, psychopathology, functioning, baseline exposure to psychiatric treatments in the whole sample and subgroups

Characteristic	Total (N = 116)	non-CHR-P (N = 50)	CHR-P (N = 47)	Psychosis (N = 19)	p
Personal history of any DSM-5 psychiatric disorder					
Number of DSM-5 diagnoses, mean ± SD	1.62 ± 1.4	1.5 ± 0.6	1.8 ± 0.7	1.4 ± 0.7	.039*
Number of diagnoses ≥ 3, n(%)	9(7.8)	2(4.0)	6(12.8)	1(5.3)	0.247
Onset of psychiatric symptoms, months, median (IQR25,75)	18.0(8.0,48.0)	21.0(8.0, 60.0)	18.0(8.0, 48.0)	18.0(9.0, 48.0)	0.94
Type of DSM-5 diagnoses, n(%)					
Depressive disorders	43(37.1)	18(36.0)	21(44.7)	4(21.1)	0.155
Anxiety disorders	28(24.1)	13(26.0)	13(27.7)	2(10.5)	0.273
Personality disorders	25(21.6)	9(18.0)	15(31.9)	1(5.3)	.035*
Disruptive, impulse-control, and conduct disorders	7(6.0)	3(6.0)	2(4.3)	2(10.5)	0.656
Eating disorders	15(12.9)	6(12.0)	8(17.0)	1(5.3)	0.394
Bipolar symptoms	6(5.2)	0.0(0.0)	6(12.8)	0(0.0)	.009*
Conversion disorder	5(4.3)	2(4.0)	3(6.4)	0 (0)	0.494
Obsessive–compulsive and related disorders	4(3.4)	1(2.0)	2(4.3)	1(5.3)	0.753
Others^a^	26(22.4)	12(24.0)	6(12.8)	8(42.1)	.042*
Specific psychiatric disorders					
* Depressive disorders*					0.330
Major depressive disorder	15(12.9)	5(10)	8(17.0)	2.0(10.5)	
Other specified depressive disorder	25(21.6)	13(26.0)	10(21.3)	2(10.5)	
Persistent depressive disorder	2(1.7)	0(0.0)	2(4.3)	0(0.0)	
* Anxiety disorders*					0.499
Generalized anxiety disorder	5(4.3)	1(2.0)	3(6.4)	1(5.3)	
Social anxiety disorder	3(2.6)	1(2.0)	1(2.1)	1(5.3)	
Other specified anxiety disorder	11(9.5)	7(14.0)	4(8.5)	0(0)	
Separation anxiety disorder	0(0.0)	0(0.0)	0(0.0)	0(0)	
Panic disorder	6(5.2)	2(4.0)	4(8.5)	0(0)	
* Personality disorders (PD)*					0.116
Borderline	11(9.5)	3(6.0)	7(14.9)	1(5.3)	
Others PD^b^	14(12.1)	6(12.0)	8(17.0)	0(0)	
* Eating disorders*					0.140
Anorexia nervosa	10(8.6)	2(4.0)	7(14.9)	1(5.3)	
Others (bulimia/binge eating)	5(4.3)	4(8.0)	1(2.1)	0 (0)	
* Bipolar symptoms*					0.099
Bipolar I or II symptoms	4(3.4)	0(0.0)	4(8.5)	0(0)	
Other specified bipolar symptoms	1(0.9)	0(0.0)	1(2.1)	0(0)	
* Psychosis*					
Psychosis	5 (4.31)	1 (2.0) ^c^	0 (0)	4 (21.05)	
APS	47 (40.52)	0 (0)	47 (100)	0 (0)	
Other psychotic disorder	14 (12.07)	5 (10.0)	0 (0)	9 (47.37)	
Presence of negative symptoms, n (%)	90(77.6)	33(66.0)	39(83.0)	18(94.7)	.020*
CAARMS median (IRQ 25,75)					
P1. Unusual thought content					
Severity	1.0(0.0,4.0)	0.0(0.0,0.75)	2.0(1.0, 4.0)	5.0(2.0,5.0)	< .001**
Frequency	2.0(0.0,4.0)	0.0 (0.0,0.75)	3.0(1.5, 4.5)	5.0(3.0,5.0)	< .001**
P2. Non-bizarre ideas					
Severity	2.0(0.0, 4.0)	0.0(0.0,2.0)	3.0(2.0, 4.0)	5.0(5.0,6.0)	< .001**
Frequency	3.0(0.0, 5.0)	0.0(0.0,2.0)	4.0(1.5, 5.0)	5.0 (5.0, 6.0)	< .001**
P3. Perceptual abnormalities					
Severity	3.0(0.0,4.0)	0.0(0.0,2.0)	3.0(2.0,4.0)	5.0(4.0,5.0)	< .001**
Frequency	2.0(0.0,4.0)	0.0(0.0,1.7)	3.0(1.0,4.0)	4.0(3.0,5.0)	< .001**
P4. Disorganized speech					
Severity	1.5(0,3.0)	0.0(0.0,0.7)	2.0(0.0,3.0)	3.0 (2.0,5.0)	< .001**
Frequency	1.0(0.0,4.0)	0.0(0.0,1.0)	3.0(0.0,4.0)	5.0(3.0,6.0)	< .001**
Clinical Global Impression-Severity (CGI-S) median (IRQ 25, 75)	4.0 (3.0, 6.0)	3.0 (3.0, 4.0)	5.0 (4.0, 6.0)	6.0 (6.0, 6.0)	< .001**
*Functioning*					
Current SOFAS median (IRQ 25,75)	51.0 (40.0,60.0)	60.0 (55.0,70.0)	50.0 (40.0,53.0)	32.0 (30.0,41.0)	<.001**
Current role functioning (GF:R) median (IRQ 25,75)	5.0 (3.0, 6.0)	6.0 (5.0,7.0)	4.0 (3.0,6.0)	3.0 (2.0,3.0)	< .001**
Current social functioning (GF:S) median (IRQ 25,75)	5.0 (3.0,7.0)	6.0 (5.0,7.75)	5.0 (3.0,6.0)	3.0 (2.0,4.0)	< .001**
Global assessment functioning (CGAS)	50.0 (40.0,60.0)	60.0 (51.0,70.0)	50.0 (41.0,50.0)	35.0 (30.0,40.0)	< .001**
*Before baseline exposure to psychiatric treatments*					
Psychotropic drugs, yes, n (%)	46 (39.7)	11 (22.0)	22 (46.0)	13 (86.0)	< .001**
Number of psychotropic drugs, median (min, max)	0.0 (0.0,4.0)	0.0 (0.0,3.0)	0.0 (0.0,4.0)	1.0 (0.0,3.0)	0.11
Type of psychotropic drugs, n (%)					
Antipsychotics	23 (19.8)	4 (8.0)	10 (21.3)	9 (47.4)	.002*
Antidepressants	22 (19.0)	7 (14.0)	11 (23.4)	4 (21.1)	0.511
Benzodiazepines	23 (19.8)	3 (6.0)	13 (27.7)	7 (36.8)	.005*
Mood stabilizers	4.0 (3.4)	1 (2.0)	2 (4.3)	1 (5.3)	0.765
Duration of psychotropic treatment, days, median (IQR 25, 75)	0.0 (0.0,30.0)	0.0 (0.0,0.0)	0.0 (0.0,6.0)	2.0 (0.0,24.0)	.009*
* Before baseline exposure to psychiatric treatments*					
Drugs prescription during baseline, yes, n (%)	72 (62.1)	19 (38.0)	36 (76.6)	17 (89.5)	< .001**
Antipsychotics	41 (35.3)	7 (14.0)	18 (38.3)	16 (84.2)	< .001**
Antidepressants	44 (37.9)	14 (28.0)	22 (46.8)	8 (42.1)	0.168
Benzodiazepines	26 (22.4)	6 (12.0)	12 (25.5)	8 (42.1)	.031*
Mood stabilizers	7 (6.0)	1 (2.0)	4 (8.5)	1 (10.5)	0.291
Psychotherapy, yes, n (%)	51 (44.0)	20 (40.0)	23 (48.9)	8 (42.1)	0.666
Psychotherapy duration, days, median (IQR 25, 75)	0.0 (0.0,12.0)	0.0 (0.0,10.0)	1.0 (0.0, 10.5)	0.0 (0.0,12.0)	0.960

*CHR-P* clinical high risk for psychosis

^a^Attention Deficit Hyperactivity Disorders, Tics, Post-traumatic disorders/adjustment

^b^Avoidant, dependent, narcissistic, schizotypal, other specified personal disorders

^c^In remission

The three groups (i.e., psychosis, CHR-P, non-CHR-P) did not differ in terms of age H (2) = 1.398, *p* = 0.49; gender, H (2) = 1.670, *p* = 0.43; SES, H (2) = 4.796, *p* = 0.78; or ethnicity, H (2) = 2.822, *p* = 0.24.

### Neurocognition

Table 3 show between-groups comparisons of IQ dimensions, neurocognitive tasks, and post-hoc analyses. Results revealed significant differences in the working memory performance and processing speed subtests of the Wechsler scale between adolescents from psychosis and non-CHR-P groups, showing psychotic adolescents perform worse than the non-CHR-P ones. Focusing on neuropsychological domains, adolescents from the psychosis group significantly differed from the CHR-P and non-CHR-P group in TMT-A, indicating a lower performance, BVN categorical fluency, revealing more inadequate flexibility skills. Psychotic adolescents also had a lower performance in BVN forward and backward verbal digit span and visual attention than Non-CHR-P adolescents and worse performance in Rey–Osterrieth complex figure test than CHR-P adolescents.

**Table 3:** Comparisons of IQ dimensions and neurocognitive tasks, and post-hoc analyses

		Non-CHR-P *N* = 50		CHR-P *N* = 47		Psychosis *N* = 19				Non-CHR-P vs CHR-P	CHR-P vs Psychosis	Non-CHR-P vs Psychosis
		*M*	*SD*	*M*	*SD*	*M*	*SD*	χ^2^	*p*	*p*	*p*	*p*
Wechsler scale	Full scale IQ	101.33	17.895	98.47	16.367	91.58	14.535	4.089	0.129			
	VCI	104.02	18.503	103.62	18.184	102.26	16.931	0.425	0.809			
	PRI	103.38	17.308	104.20	16.280	98.89	15.376	1.132	0.568			
	WMI	94.58	14.094	91.38	13.202	84.89	11.818	6.430	.040*	0.470	0.051	.012*
	PSI	98.58	16.354	93.82	15.686	82.68	15.567	10.686	.005*	0.177	.027*	.001**
TMT	TMT A	34.87	14.385	34.71	10.509	47.89	14.476	12.679	.002*	0.601	.002*	< .001**
	TMT B	74.79	26.899	77.67	36.662	96.53	62.180	3.975	0.137			
	TMT B-A	39.88	21.825	44.78	29.460	58.32	46.386	3.216	0.200			
BVN 12–18	Lexical Denomination	89.575	32.721	90.718	14.796	84.379	19.907	3.590	0.166			
	Forward verbal digit span	93.929	14.038	90.673	18.895	84.311	8.365	8.394	.15*	0.469	.022*	.004*
	Backward verbal digit span	99.502	17.606	93.407	12.401	89.432	13.575	6.650	.036*	0.164	0.146	.011*
	Corsi Block-tapping test	99.079	17.519	92.021	21.672	90.197	19.117	3.035	0.219			
	Selective auditory	85.217	26.391	74.838	41.761	75.974	31.133	2.760	0.252			
	Visual attention	109.187	14.590	107.896	14.331	93.874	28.510	7.984	.018*	0.469	.026*	.005*
	Phonemic fluency	100.604	19.661	102.031	18.224	94.689	26.958	2.937	0.230			
	Categorial fluency	95.542	19.110	95.584	15.670	81.768	16.946	8.714	.013*	0.914	.006*	.007*
	Elithorn perceptual maze	97.985	20.333	93.673	25.238	80.605	31.616	2.862	0.239			
ROCF	Copy	32.630	4.170	32.468	3.580	30.579	4.605	4.72	0.94			
	Drawing from memory	22.100	6.434	22.521	6.994	17.289	6.449	8.57	.014*	0.753	.005*	.009*

*BVN 12–18* adolescent neuropsychological assessment battery, *PRI* perceptual reasoning index, *PSI* processing speed index, *ROCF* Rey–Osterrieth complex figure test, *TMT* trail making test, *VCI* verbal comprehension index, *WMI* working memory index

^*^p < .05

**p < .001

### Functioning

Results showed the CHR-P group to have a more adaptive functioning (e.g., SOFAS, GF:R, GF:S, and CGAS) than the psychosis group but worse functioning than the non-CHR-P group on all the scales. We also found that the CHR-P group presented a lower CGI-S level than the psychosis group but higher than the non-CHR-P one, as shown in Table 2.

## Discussion

This work highlighted significant differences between the three groups of patients in neurocognition and functioning. However, they did not differ in age, gender, socio-economic status, ethnicity, adoption, separated/divorced parents, or history of family psychiatric disorders. Regarding neurocognitive functioning, the CHR-P group performed better than the psychosis group on the working memory and backward verbal digit span tasks, as previous research suggested [14, 16]. Results in the adult population showed that the CHR-P group could be distinguished from the psychosis group using verbal learning tasks, since the latter group seem to perform worse [9]. This could be explained because language development is still evolving in adolescents; at this stage of life, they learn to think abstractly and develop the use of pragmatics and semantics. Therefore, language-related difficulties may be more evident in an adult population sample. Moreover, the difference between our data and adults and adolescent-adult samples may be explained by possible biases due to the greater presence of females in our sample that may have created a bias given the higher prevalence of psychotic onset in the male population. Literature states that psychosis typically onsets in adolescence and early adulthood [1] and much research has highlighted the importance of detection, prognosis, and interventions for improving the outcomes of CHR-P people because it is challenging to lead the patient to complete recovery from psychosis [2, 3]. Despite childhood and adolescence representing a complex developmental phase studies in this population are few [12–17, 48] as it is challenging to investigate neurocognition in young patients. This is one of the few works that explored this domain.

Furthermore, our data did not show substantial differences in neurocognition between CHR-P and non-CHR-P patients’ performances, maybe because our non-CHR-P sample was composed of patients who presented other psychiatric symptoms without psychotic symptoms and were not healthy controls. Likewise, our results did not match those found among adults between CHR-P patients and healthy controls, which see the CHR-P group performing worse in every neurocognitive task, maybe because the adolescent brain goes through a critical developmental period of increased neural plasticity, unlike adults, and this may also reflect the greater number of comorbidities in our patient sample [9, 49]. Moreover, as previous literature stated [50], we should consider adolescents as a more heterogeneous group than adults, and we have to think in terms of a developmental psychopathology perspective, not only to deepen the knowledge of adolescent psychopathology but also to understand developmental processes more generally [1].

In line with previous literature [14–16], patients in the psychosis group compared to the non-CHR-P group, exhibited significant deficits in working memory, processing speed, forward verbal digit span, backward verbal digit span, visual attention, categorical fluency, executive functions, psychomotor speed, and visuospatial attention and planning tasks.

As for the overall functioning, the CHR-P group exhibited better global functioning, better role and social functioning than the psychosis group, but still worse functioning than the non-CHR-P group [51]. Moreover, the CHR-P group showed a more significant presence of diagnoses of structuring personality disorder and bipolar symptoms. This group has many diagnoses of eating disorders [52, 53]. In line with the literature [16], we found that the psychosis group had a massive presence of severe positive and negative symptoms compared to the other groups and was also the group with the lowest global functioning, the most compromised role and social functioning, and the most severe level of disorder severity based on clinical evaluation.

The study has some limitations. Future studies could consider a larger sample of adolescent patients or even younger participants to study the possibility of increasingly early prevention of developing psychotic symptoms. Furthermore, researchers could select different neuropsychological tests to identify better areas that do not show a significant difference in our population sample (e.g., problem-solving, comprehension tasks, Theory of Mind). Finally, our results could be implemented by including a longitudinal study phase that could document transition rates.

These results examining a population understudied contribute to making the assessment more rigorous, and specific functional and neurocognitive impairments can be a prognostic biomarker in identifying particular groups of patients, even in a developmentally complex period such as adolescence, and recommending the most appropriate course of treatment and preparing, where necessary, prevention pathways, as many studies over the years have pointed out [9, 17, 54–58]. Moreover, given the not consistently overlapping results [9], this research opens up new studies to standardize the assessment and to better detect the risk of transition to psychosis.

## Supplementary Information

**Additional file 1.** The Reporting of studies Conducted using the Observational Routinely collected health Data(RECORD) statement.

**Additional file 2. Table S1.** Post-Hoc adjusted p-values**. **Table S2. Comprehensive Assessment of At risk Mental State (CAARMS) nondiagnostic subscale in the three groups.

## Data Availability

The dataset generated and analysed during the current study is available upon request in the Zenodo repository [20] at 10.5281zenodo.6325531.
