# Supplementary material for: Neurocognition and functioning in adolescents at clinical high risk for psychosis
Source: Child Adolesc Psychiatry Ment Health. 2023 Feb 8;17:22. doi: 10.1186/s13034-023-00567-1 (PMC9909975; doi:10.1186/s13034-023-00567-1)
Supplement: Supplementary file 2 — Additional file 2. Table S1. Post-Hoc adjusted p-values. Table S2. Comprehensive Assessment of At risk Mental State (CAARMS) nondiagnostic subscale in the three groups. [file 13034_2023_567_MOESM2_ESM.docx]

# Table S1. Post-Hoc adjusted p-values

| **Characteristic** | **Post-hoc adjusted p-values** | | |
| --- | --- | --- | --- |
|  | **Non-CHR-P vs CHR-P** | **Non-CHR-P vs psychosis** | **CHR-P vs Psychosis** |
|  |  |  |  |
| ***Personal history of any psychiatric disorder*** | | | |
| Number of DSM-5 diagnoses | .038 | .514 | .033 |
| Number of diagnoses ≥3 | .117 | .818 | .370 |
| Presence of negative symptoms | .056 | .022 | .208 |
| Depressive disorders | .371 | .196 | .055 |
| Anxiety disorders | .846 | .143 | .113 |
| Personality disorders | .108 | .163 | .018 |
| Bipolar symptoms (mania/hypomania) | .009 | .366 | .095 |
| Obsessive-compulsive and related disorders | .520 | .491 | .887 |
| ***Psychopathological assessment*** | | | |
| **CAARMS** | | | |
| **Unusual thought content** | | | |
| Severity | <.001 | <.001 | .005 |
| Frequency | <.001 | <.001 | .066 |
| **Non-bizarre ideas** |  |  |  |
| Severity | <.001 | <.001 | <.001 |
| Frequency | <.001 | <.001 | <.001 |
| **Perceptual abnormalities** | | | |
| Severity | <.001 | <.001 | <.001 |
| Frequency | <.001 | <.001 | .018 |
| **Disorganized speech** | | | |
| Severity | <.001 | <.001 | .014 |
| Frequency | <.001 | <.001 | .004 |
| Functioning | | | |
| Clinical Global Impression-Severity (CGI-S) | <.001 | <.001 | <.001 |
| Current SOFAS | <.001 | <.001 | <.001 |
| Current role functioning (GF:R) | <.001 | <.001 | <.001 |
| Current social functioning (GF:S) | <.001 | <.001 | <.001 |
| Global assessment functioning (CGAS) | <.001 | <.001 | <.001 |
| ***Baseline exposure to psychiatric treatments*** | | | |
| Antipsychotics | .067 | .044 | <.001 |

# Table S2. Comprehensive Assessment of At risk Mental State (CAARMS) nondiagnostic subscale in the three groups

| **Characteristic, median**  **(IQR25, 75)** | **Total (N=116)** | **non-CHR-P (N=50)** | **CHR-P (N=47)** | **Psychosis (N=19)** | **p-value** | **Post-hoc adjusted p-values** | | |
| --- | --- | --- | --- | --- | --- | --- | --- | --- |
|  |  |  |  |  |  | **Non-CHR-P vs CHR-P** | **Non-CHR-P vs psychosis** | **CHR-P vs psychosis** |
| ***Cognitive change- attention/concentration*** | | | | | | | | |
| Subjective experience (Huber’s basic symptom) | | | | | | | | |
| Severity | 2.0(0.0,3.0) | 1.5(0.0,2.0) | 2.0(2.0,3.0) | 3.0(2.0,4.0) | <.001 | <.001 | <.001 | .144 |
| Frequency | 3.0(0.0,4.0) | 1.5(0.0,4.0) | 3.0 (2.0,4.5) | 30(3.0,5.0) | .009 | .011 | .011 | .552 |
| Observed cognitive change |  |  |  |  |  |  |  |  |
| Severity | 0.0(0.0,2.0) | 0.0(0.0,0.0) | 0.0(0.0,2.0) | 2.0(0.0,4.0) | <.001 | .009 | <.001 | .039 |
| ***Emotional disturbances*** | | | | | | | | |
| Subjective emotional disturbance (Huber’s basic symptom) | | | | | | | | |
| Severity | 2.0(0.0,3.0) | 0(0.0,2.0) | 3.0(2.0,4.0) | 3.0(0.0, 4.0) | <.001 | <.001 | .002 | .229 |
| Frequency | 3.0 (0.0,4.0) | 0.0(0.0,3.0) | 4.0(3.0,5.0) | 3.0(0.0, 4.0) | <.001 | <.001 | .044 | .080 |
| Observed blunted affect | | | | | |  | | |
| Severity | 2.0(0.0,3.0) | 0.0(0.0,3.0) | 3.0(0.5,3.0) | 3.0(0.0,3.0) | .007 | .007 | .014 | .703 |
| Frequency | 3.0(0.0,4.0) | 0.0(0.0,3.0) | 3.0(0.0,4.0) | 3.0(0.0,5.0) | .008 | .015 | .007 | .409 |
| Observed inappropriate affect | | | | | | | | |
| Severity | 0.0(0.0,3.0) | 0.0(0.0,0.0) | 2.0(0.0,3.0) | 0.0(0.0,3.0) | <.001 | <.001 | .007 | .999 |
| Frequency | 0.0(0.0,3.0) | 0.0(0.0,0.0) | 3.0(0.0,3.0) | 0.0(0.0, 4.0) | <.001 | <.001 | .007 | .962 |
| ***Negative symptoms*** | | | | | | | | |
| Alogia | | | | | | | | |
| Severity | 0.0(0.0,3.0) | 0.0(0.0,1.0) | 2.0(0.0,3.0) | 3.0(0.0, 3.0) | <.001 | .002 | <.001 | .151 |
| Frequency | 0.0(0.0,3.75) | 0.0(0.0,1.0) | 3.0(0.0,4.0) | 3.0(0.0,5.0) | <.001 | .003 | <.001 | .237 |
| Avolition/Apathy (Huber’s basic symptom) | | | | | | | | |
| Severity | 3.0(2.0,4.0) | 3.0(0.25,3.75) | 4.0(2.0,4.0) | 4.0(2.0,5.0) | .004 | .006 | .005 | .469 |
| Frequency | 4.0 (2.0,5.0) | 3.0(0.0,4.75) | 5.0(3.0, 5.5) | 5.0(4.0,6.0) | <.001 | <.001 | <.001 | .437 |
| Anhedonia |  |  |  |  |  |  |  |  |
| Severity | 2.0(0.0,4.0) | 0.0(0.0,3.0) | 2.0(0.0,4.0) | 4.0(0.0,5.0) | .048 | .071 | .026 | .407 |
| Frequency | 3.5(0.0,5.0) | 0.0(0.0,4.0) | 3.0(0.0,5.0) | 4.0(0.0,5.0) | .051 |  |  |  |
| ***Behavioral change*** | | | | | | | | |
| Social isolation | | | | | | | | |
| Severity | 3.0(1.0,4.0) | 2.0(0.0,3.0) | 3.0(2.0,4.0) | 4.0(4.0, 4.0) | <.001 | .004 | <.001 | .014 |
| Frequency | 4.0(1.0,4.0) | 3.0(0.0,4.0) | 4.0(3.0,5.0) | 5.0(4.0,6.0) | <.001 | .003 | <.001 | .016 |
| Impaired role function | | | | | | | | |
| Severity | 3.0(0.0,4.0) | 2.0(0.0,3.0) | 3.0(2.0,5.0) | 4.0(4.0,5.0) | <.001 | .002 | <.001 | .048 |
| Frequency | 4.0(0.5,5.0) | 3.0(0.0,4.0) | 4.0(3.0,5.0) | 5.0(4.0, 6.0) | <.001 | <.001 | <.001 | .082 |
| Disorganized/odd/stigmatizing behavior | | | | | | | | |
| Severity | 0.0(0.0,3.0) | 0.0(0.0,0.0) | 1.0 (0.0,3.0) | 3.0(0.0,5.0) | <.001 | .002 | <.001 | .157 |
| Frequency | 0.0(0.0,3.0) | 0.0(0.0,0.0) | 2.0(0.0,3.0) | 4.0(0.0,5.0) | <.001 | .004 | <.001 | .212 |
| Aggressive/dangerous behavior | | | | | | | | |
| Severity | 3.0(2.0,4.0) | 2.0(0.0,3.0) | 3.0(2.0,4.0) | 4.0(3.0,4.0) | .018 | .073 | .007 | .190 |
| Frequency | 3.0(2.0,4.0) | 3.0(0.0,4.0) | 3.0(2.0,4.0) | 4.0(3.0,5.0) | .027 | .105 | .010 | .182 |
| *Motor/physical changes* | | | | | | | | |
| Subjective complaints of impaired motor functioning (Huber’s basic symptom) | | | | | | | | |
| Severity | 0.0(0.0,0.0) | 0.0(0.0,0.0) | 0.0(0.0,2.0) | 0.0(0.0,2.0) | .018 | .091 | .006 | .684 |
| Frequency | 0.0(0.0,0.0) | 0.0(0.0,0.0) | 0.0(0.0,2.0) | 0.0(0.0,4.0) | .011 | .086 | .003 | .599 |
| Informant reported or observed changes in motor functioning | | | | | | | | |
| Severity | 0.0(0.0,0.0) | 0.0(0.0,0.0) | 0.0(0.0,0.0) | 0.0(0.0,3.0) | <.001 | .342 | <.001 | .003 |
| Subjective complaints of impaired bodily sensation (Huber’s basic symptom) | | | | | | | | |
| Severity | 0.0(0.0,2.0) | 0.0(0.0,0.0) | 0.0(0.0,2.5) | 0.0(0.0,3.0) | <.001 | .027 | <.001 | .548 |
| Frequency | 0.0(0.0,2.0) | 0.0(0.0,0.0) | 0.0(0.0,3.0) | 0.0(0.0,3.0) | 0.001 | .033 | <.002 | .413 |
| Subjective complaints of impaired autonomic functioning (Huber’s basic symptom) | | | | | | | | |
| Severity | 2.0(0.0,3.0) | 2.0(0.0,3.0) | 2.0(0.0,3.0) | 2.0(0.0,3.0) | .211 |  |  |  |
| Frequency | 2.0(0.0,4.0) | 2.0(0.0,3.0) | 3.0(0.0,4.5) | 2.0(0.0,3.0) | .080 |  |  |  |
| ***General psychopathology*** | | | | | | | | |
| Mania | | | | | | | | |
| Severity | 0.0(0.0,2.0) | 3.0(0.0, 2.0) | 0.0(0.0,2.5) | 0.0(0.0,0.0) | .425 |  |  |  |
| Frequency | 0.0(0.0,2.0) | 3.5(0.0, 2.0) | 0.0(0.0,3.0) | 0.0(0.0,0.0) | .140 |  |  |  |
| Depression | | | | | | | | |
| Severity | 3.0(2.0,4.0) | 3.0(2.0,4.0) | 4.0(2.0,5.0) | 4.03.0, 5.0,4.0) | .024 | .025 | .010 | .390 |
| Frequency | 4.0(3.0,5.0) | 3.5(2.0,5.0) | 5.0 (3.0,5.0) | 5.0(3.0,5.0) | <.001 | .012 | .051 | .970 |
| Suicidality and self–harm | | | | | | | | |
| Severity | 2.0(0.0,3.0) | 0.0(0.0,2.0) | 3.0(0.0,4.0) | 3.0(0.0,4.0) | <.001 | <.001 | .003 | .717 |
| Frequency | 1.0(0.0,3.0) | 0.0(0.0,2.0) | 3.0(0.0,4.0) | 3.0 (0.0,4.0) | <.001 | .013 | <.001 | .742 |
| Mood swings/lability | | | | | | | | |
| Severity | 1.5(0.0,3.0) | 1.0(0.0,2.75) | 2.0(0.0,3.0) | 0.0(0.0,3.0) | .391 |  |  |  |
| Frequency | 1.0(0.0,3.0) | 0.5(0.0,3.0) | 3.0(0.0,4.0) | 0.0(0.0,3.0) | .229 |  |  |  |
| Anxiety | | | | | | | | |
| Severity | 3.0(3.0,4.0) | 3.0 (1.25,4.0) | 4.0(3.0, 5.0) | 4.0(3.0,5.0) | .006 | .009 | .006 | .464 |
| Frequency | 4.0(2.0,5.0) | 3.0(1.0,4.0) | 4.0(2.0,5.0) | 4.0(3.0,5.0) | .005 | .004 | .012 | .772 |
| OCD symptoms | | | | | | | | |
| Severity | 0.0(0.0,2.0) | 0.0(0.0,2.0) | 1.0(0.0,2.0) | 0.0(0.0,3.0) | .224 |  |  |  |
| Frequency | 0.0(0.0,4.0) | 0.0(0.0,2.0) | 1.0(0.0,4.0) | 0.0(0.0,4.0) | 153 |  |  |  |
| Dissociative symptoms | | | | | | | | |
| Severity | 0.0(0.0,2.0) | 0.0(0.0,0.0) | 0.0(0.0,3.0) | 2.0(0.0,2.0) | .002 | .003 | .003 | .562 |
| Frequency | 0.0(0.0,2.0) | 0.0(0.0,0.0) | 0.0(0.0,3.0) | 1.0(0.0,3.0) | <.001 | <.001 | .002 | .544 |
| Impaired tolerance to normal stress (Huber’s basic symptom) | | | | | | | | |
| Severity | 3.0(0.0,4.0) | 2.0(0.0,4.0) | 3.0(0.0,4.0) | 4.0(0.0,4.0) | .174 |  |  |  |
| Frequency | 3.0(0.0,4.0) | 2.0(0.0,4.0) | 3.0(0.0,5.0) | 3.0(0.0,5.0) | .145 |  |  |  |
